# Amalgam Fillings

**Published:** 1872-11

**Authors:** H. Scott

**Affiliations:** Lancaster, Ohio


### Amalgam Fillings.

BY. H. SCOTT, LANCASTER, OHIO.

I have come to have a high appreciation of amalgams as cheap fillings in some cases; but their value depends entirely upon, first, the manipulation of the metal, and secondly, the manner of putting it *in situ.* It occurs, I suppose, to every dentist to see, from time to time, execrably bad amalgam fillings; and that which I want to say is, that I know that the failures are always the result of the want of pains-taking in doing the work. There are cases always presenting where the patient is neither able nor willing to pay the price that must be charged for gold work. And further, there are cases where I recommend amalgam. Back teeth whose parieties or general structure is too frail to bear the force of consolidating the gold, or to promise to remain a long time. In front teeth I never use amalgam in any case, preferring tin, if cheap fillings must be had. My method of using amalgam may suffice for what I want to teach.

The same care in the preparation of cavities as if gold were to be used is of course necessary. I triturate my mass in a mortar two or three minutes before applying the wash, and then continue the trituration for at least double the time, changing the wash until it is no longer colored. I then take the mass from the mortar, with the point of the middle finger, and place it on a folded muslin or linen napkin, which I turn over it and press out all the moisture. Then, everything being ready, I squeeze the excess of mercury out through the chamois skin, until no more can be forced out. Then cut the metal in suitable sizes, dry the cavity thoroughly, and commence packing. I deem it important that the moisture should be thoroughly removed, and that the metal should be plastered over the entire walls of the cavity to the margin, before the damp is allowed to reach it. After that I think it is no detriment to the work if the tooth becomes submerged with saliva. I pack the metal firmly to every corner and retaining point, and spend a great deal of time in speaking. It is impossible to pack too much. After the work has stood an hour or so, I pack over the work again, and rub away the soft part that comes on the surface. Leave the surface as smooth as possible, and in a day or two polish.

This is the entire secret of success in amalgam work; a great deal of labor and pains-taking. Fillings thus made and finely polished will retain their silver luster, and wear a lifetime, and show no appreciable contraction from the margins of the cavities, with the use of even powerful glasses. I use Lawrence's preparation, but believe others as good. No man can succeed by mixing in his hand.

